# Machine learning-based prediction of clinical outcomes after first-ever ischemic stroke

**DOI:** 10.3389/fneur.2023.1114360

**Published:** 2023-02-21

**Authors:** Lea Fast, Uchralt Temuulen, Kersten Villringer, Anna Kufner, Huma Fatima Ali, Eberhard Siebert, Shufan Huo, Sophie K. Piper, Pia Sophie Sperber, Thomas Liman, Matthias Endres, Kerstin Ritter

**Affiliations:** ^1^Charité - Universitätsmedizin Berlin, Corporate Member of Freie Universität Berlin and Humboldt-Universität zu Berlin, Department of Psychiatry and Psychotherapy, Berlin, Germany; ^2^Charité - Universitätsmedizin Berlin, Corporate Member of Freie Universität Berlin and Humboldt-Universität zu Berlin, Center for Stroke Research Berlin (CSB), Berlin, Germany; ^3^Berlin Institute of Health at Charité - Universitätsmedizin Berlin, Berlin, Germany; ^4^Charité - Universitätsmedizin Berlin, Corporate Member of Freie Universität Berlin and Humboldt-Universität zu Berlin, Department of Neurology with Experimental Neurology, Berlin, Germany; ^5^Berlin School of Mind and Brain, Humboldt-Universität zu Berlin, Berlin, Germany; ^6^Charité - Universitätsmedizin Berlin, Corporate Member of Freie Universität Berlin and Humboldt-Universität zu Berlin, Department of Neuroradiology, Berlin, Germany; ^7^German Center for Cardiovascular Research (Deutsches Zentrum für Herz-Kreislauferkrankungen, DZHK), Partner Site Berlin, Berlin, Germany; ^8^Charité - Universitätsmedizin Berlin, Corporate Member of Freie Universität Berlin and Humboldt-Universität zu Berlin, Institute of Biometry and Clinical Epidemiology, Berlin, Germany; ^9^Charité - Universitätsmedizin Berlin, Corporate Member of Freie Universität Berlin and Humboldt-Universität zu Berlin, Institute of Medical Informatics, Berlin, Germany; ^10^Charité - Universitätsmedizin Berlin, Corporate Member of Freie Universität Berlin and Humboldt-Universität zu Berlin, NeuroCure Cluster of Excellence, NeuroCure Clinical Research Center (NCRC), Berlin, Germany; ^11^Experimental and Clinical Research Center, A Cooperation Between the Max Delbrück Center for Molecular Medicine in the Helmholtz Association and Charité – Universitätsmedizin Berlin, Berlin, Germany; ^12^Max Delbrück Center for Molecular Medicine in the Helmholtz Association (MDC), Berlin, Germany; ^13^German Center for Neurodegenerative Diseases (Deutsches Zentrum für Neurodegenerative Erkrankungen, DZNE), Partner Site Berlin, Berlin, Germany; ^14^Department of Neurology, Evangelical Hospital Oldenburg, Carl von Ossietzky-University, Oldenburg, Germany; ^15^Charité - Universitätsmedizin Berlin, Corporate Member of Freie Universität Berlin and Humboldt-Universität zu Berlin, Bernstein Center for Computational Neuroscience (BCCN), Berlin, Germany

**Keywords:** stroke, machine learning, outcome prediction, post-stroke depression, mortality, functional outcome, cognitive impairment

## Abstract

**Background:**

Accurate prediction of clinical outcomes in individual patients following acute stroke is vital for healthcare providers to optimize treatment strategies and plan further patient care. Here, we use advanced machine learning (ML) techniques to systematically compare the prediction of functional recovery, cognitive function, depression, and mortality of first-ever ischemic stroke patients and to identify the leading prognostic factors.

**Methods:**

We predicted clinical outcomes for 307 patients (151 females, 156 males; 68 ± 14 years) from the PROSpective Cohort with Incident Stroke Berlin study using 43 baseline features. Outcomes included modified Rankin Scale (mRS), Barthel Index (BI), Mini-Mental State Examination (MMSE), Modified Telephone Interview for Cognitive Status (TICS-M), Center for Epidemiologic Studies Depression Scale (CES-D) and survival. The ML models included a Support Vector Machine with a linear kernel and a radial basis function kernel as well as a Gradient Boosting Classifier based on repeated 5-fold nested cross-validation. The leading prognostic features were identified using Shapley additive explanations.

**Results:**

The ML models achieved significant prediction performance for mRS at patient discharge and after 1 year, BI and MMSE at patient discharge, TICS-M after 1 and 3 years and CES-D after 1 year. Additionally, we showed that National Institutes of Health Stroke Scale (NIHSS) was the top predictor for most functional recovery outcomes as well as education for cognitive function and depression.

**Conclusion:**

Our machine learning analysis successfully demonstrated the ability to predict clinical outcomes after first-ever ischemic stroke and identified the leading prognostic factors that contribute to this prediction.

## 1. Introduction

Stroke is the second most common cause of death and a major cause of disability on a worldwide scale (1). It occurs when the blood supply to brain tissue is interrupted by either blockage (ischaemic stroke) or bleeding caused by rupture of cerebral blood vessels (haemorrhagic stroke) ultimately resulting in irreversible neuronal death (2). The incidence of stroke is set to rise due to the demographic shift affecting populations across the globe (3). Thus, it is paramount to identify parameters that can aid in accurate prediction of long-term clinical outcome post-stroke.

In recent years the move toward electronic health records and the application of machine learning (ML) techniques in the medical research field have opened new frontiers of personalized medicine and decision support. The key advantage is that—in contrast to traditional statistical analyses—not only can predictors and biomarkers be identified on a group level, but ML techniques also enable prediction on an individual patient level. In other words, the outcome for a single patients can be predicted by considering a vast array of variables (4). Numerous studies have successfully demonstrated the ability of ML models to predict specific clinical outcomes after stroke with remarkable accuracy and identified leading baseline factors that carry high prognostic value (5–8). Most studies so far have focused on the prediction of the modified Rankin Scale (mRS) (9) as it is the gold standard for determining functional recovery after stroke. While there are some studies investigating the ML-based prediction of the Barthel Index (BI) (10) and Modified Telephone Interview for Cognitive Status (TICS-M) (11), research regarding the Center for Epidemiologic Studies Depression Scale (CES-D) (12) and Mini-Mental State Examination (MMSE) (13) is sparse. In addition, the heterogeneity of ML techniques, clinical outcomes and datasets used in these studies makes it difficult to assess the broader implications of their findings (4).

The primary aim of the present study was therefore to conduct a systematic comparison of ML-based outcome prediction after first-ever ischemic stroke featuring measures of functional recovery (mRS, BI), cognitive function (MMSE, TICS-M), depression (CES-D), and mortality. The analysis was based on three powerful ML models and an array of baseline features including demographic, clinical, serological and MRI variables. As a secondary aim, we set out to identify to the key prognostic markers for each outcome using state-of-the-art visualization techniques.

## 2. Methods

### 2.1. Dataset and feature selection

The patients included in these analyses were selected from the PROSpective Cohort with Incident Stroke Berlin (PROSCIS-B) study. Recruitment for this prospective cohort study was conducted over a three-year period starting in March 2010 at the Center for Stroke Research Berlin and Charité University Hospital with a consecutive three-year follow-up period. The study population consists of patients aged 18 years and over with acute first-ever stroke according to the WHO stroke criteria (14). The complete inclusion and exclusion criteria are described in detail on https://clinicaltrials.gov (NTC01363856). The study was approved by the ethics committee of the Charité - Universitätsmedizin Berlin (EA1/218/09) and was conducted in accordance with the Declaration of Helsinki. For the purposes of this exploratory analysis only patients with ischemic stroke and input features with no more than 15% missing values were included.

MRI data was collected after study completion from clinical routine data. In order to quantify the characteristics of the imaging data all acute and chronic stroke lesions were delineated on Diffusion-weighted imaging (DWI) and Fluid-attenuated inversion recovery (FLAIR) sequences, respectively, using MRIcron (15) from the Center for Advanced Brain Imaging (University of South Carolina, Chris Rordan, USA). The delineation and volume extraction for acute and chronic stroke lesions were performed by medical students supervised by two independent expert neuroradiologists while all further MRI parameters were obtained by expert neuroradiologists.

**Figure 1:**
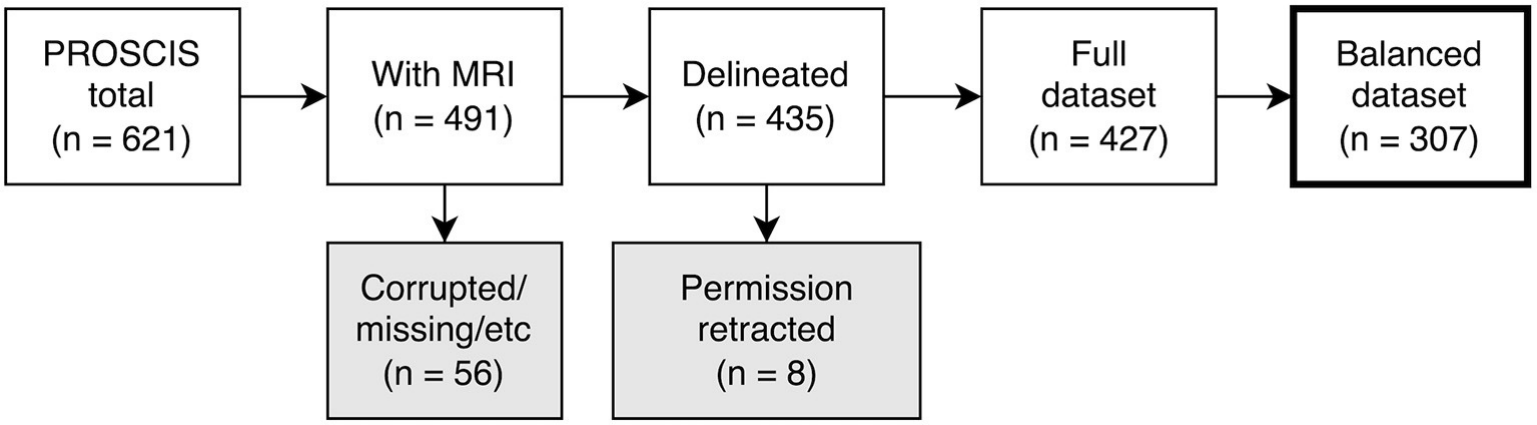
Flowchart depicting patient selection process. PROSCIS, PROSpective Cohort with Incident Stroke; MRI, Magnetic resonance imaging.

Due to significant differences in the number and mean age of female and male patients, we balanced the dataset by separating all patients into groups according to sex and age and then randomly selecting patients within these groups until there were no more significant differences (up to *p* ≤ 0.1). This was necessary to ensure the predictions of our models were not based on an inherent bias in the training data (e.g., women being older on average and thus having worse outcomes) (16). The patient selection process is shown in Figure 1 and the characteristics of the dataset are described in Table 1.

**Table 1:** Baseline characteristics of patient population.

**Parameter**		**Total *n* = 307**	**Female**	**Male**	***P*-value**
**Demographic**					
Sex, *n* (%)		307	151 (49.2)	156 (50.8)	
Age in years, Mean ± SD		68 ± 14	69 ± 15	67 ± 13	0.1
Education in years, Mean ± SD		14.1 ± 4.6	12.9 ± 4.3	15.3 ± 4.6	0.0
BMI, Mean ± SD		27.3 ± 4.8	27.5 ± 5.4	27.1 ± 4.0	0.16
Waist circumference [mm], Mean ± SD		997.4 ± 131.6	964.6 ± 142.1	1,028.1 ± 113.0	0.0
Hip circumference [mm], Mean ± SD		1,035.8 ± 112.5	1,034.7 ± 128.5	1,036.8 ± 95.8	0.58
**Clinical**					
Blood pressure, Mean ± SD	Systolic	140.1 ± 22.3	139.8 ± 22.8	140.3 ± 21.7	0.57
	Diastolic	76.1 ± 13.7	74.7 ± 13.0	77.4 ± 14.3	0.03
Alcohol consumption, *n* (%)		108 (35.2)	38 (25.2)	70 (44.9)	0.0
Smoking, *n* (%)	Active	102 (33.2)	39 (25.8)	63 (40.4)	0.1
	Never	130 (42.3)	71 (47.0)	59 (37.8)	
	Former	70 (22.8)	39 (25.8)	31 (19.9)	
Dependent before stroke, *n* (%)		39 (12.7)	22 (14.6)	17 (10.9)	0.39
Physically active, *n* (%)		101 (32.9)	44 (29.1)	57 (36.5)	0.09
Thrombolysis, *n* (%)		60 (19.5)	33 (21.9)	27 (17.3)	0.48
Revascularization, *n* (%)		5 (1.6)	0 (0.0)	5 (3.2)	0.04
NIHSS, Median [IQR]		2 [1–4]	2 [1–4]	2 [1–4]	0.41
Pre-existing conditions					
Diabetes Mellitus, *n* (%)		71 (23.1)	35 (23.2)	36 (23.1)	0.61
Hypercholesterinemia, *n* (%)		66 (21.5)	32 (21.2)	34 (21.8)	0.95
Hypertension, *n* (%)		198 (64.5)	98 (64.9)	100 (64.1)	0.98
Atrial fibrillations, *n* (%)		64 (20.8)	31 (20.5)	33 (21.2)	1.00
Angina pectoris, *n* (%)		37 (12.1)	14 (9.3)	23 (14.7)	0.18
Myocardial infarction, *n* (%)		10 (3.3)	2 (1.3)	8 (5.1)	0.33
Peripheral artery disease, *n* (%)		15 (4.9)	7 (4.6)	8 (5.1)	0.95
**Serological markers**					
Glucose [mmol/L], Mean ± SD		7.3 ± 3.2	7.6 ± 3.9	7.1 ± 2.5	0.41
HbA1c [mmol/mol], Mean ± SD		10.9 ± 15.5	12.2 ± 18.9	9.5 ± 10.7	0.21
Cholesterol [mmol/L], Mean ± SD		11.1 ± 2.7	11.3 ± 2.7	10.9 ± 2.7	0.10
HDL [mmol/L], Mean ± SD		2.9 ± 0.9	3.2 ± 0.9	2.6 ± 0.8	0.00
LDL [mmol/L], Mean ± SD		6.8 ± 2.2	6.8 ± 2.3	6.7 ± 2.1	0.46
Triglycerides [mmol/L], Mean ± SD		7.6 ± 4.8	7.0 ± 4.5	8.1 ± 5.1	0.00
Creatinine [μmol/L], Mean ± SD		82.4 ± 25.0	75.9 ± 23.1	88.6 ± 25.3	0.00
eGFR [ml/min], Mean ± SD		76.8 ± 20.6	73.5 ± 21.2	79.9 ± 19.6	0.02
hsCRP [mg/L], Mean ± SD		1.2 ± 1.9	1.3 ± 2.1	1.0 ± 1.7	0.15
White blood cells [cells/mm3], Mean ± SD		8.0 ± 2.8	8.2 ± 2.8	7.8 ± 2.8	0.47
**MRI**					
Acute infarct DWI [ml], Mean ± SD		6.1 ± 14.5	5.6 ± 11.4	6.6 ± 17.1	0.17
Acute infarct FLAIR [ml], Mean ± SD		5.0 ± 12.9	4.6 ± 10.4	5.4 ± 15.0	0.11
Infarct location, *n* (%)	Supratentorial	225 (73.3)	116 (76.8)	109 (69.9)	0.18
	Infratentorial	52 (16.9)	21 (13.9)	31 (19.9)	
	Both	30 (9.8)	14 (9.3)	16 (10.3)	
Infarct side, *n* (%)	Left	138 (45.0)	67 (44.4)	71 (45.5)	0.26
	Right	132 (43.0)	69 (45.7)	63 (40.4)	
	Both	37 (12.1)	15 (9.9)	22 (14.1)	
Unilateral infarct, *n* (%)		270 (87.9)	136 (90.1)	134 (85.9)	0.46
Chronic infarct, *n* (%)		79 (25.7)	37 (24.5)	42 (26.9)	0.90
Chronic infarct [ml], Mean ± SD		1.5 ± 2.5	1.3 ± 2.4	1.6 ± 2.6	0.34
Wahlund Score, (17) Median [IQR]		6 [3–10]	6 [3.5–11]	5 [2–8]	0.02
Infarct origin, *n* (%)	MCA	143 (46.6)	75 (49.7)	68 (43.6)	0.58
	ACA	1 (0.3)	1 (0.7)	0 (0.0)	
	PCA	17 (5.5)	10 (6.6)	7 (4.5)	
	AchA	18 (5.9)	8 (5.3)	10 (6.4)	
	Infratentorial	52 (16.9)	21 (13.9)	31 (19.9)	
	Thalamus	19 (6.2)	8 (5.3)	11 (7.1)	
	Multiple	57 (18.6)	28 (18.5)	29 (18.6)	
Infarct pattern, *n* (%)	Territorial	96 (31.3)	52 (34.4)	44 (28.2)	0.61
	Subcortical	74 (24.1)	37 (24.5)	37 (23.7)	
	Scattered	72 (23.5)	35 (23.2)	37 (23.7)	
	Lacunar	1 (0.3)	0 (0.0)	1 (0.6)	
	Infratentorial	52 (16.9)	21 (13.9)	31 (19.9)	
TOAST, *n* (%)	Large-artery	88 (28.7)	46 (30.5)	42 (26.9)	0.4
	Cardioembolism	89 (29.0)	50 (33.1)	39 (25.0)	
	Small-vessel	14 (4.6)	8 (5.3)	6 (3.8)	
	Other	40 (13.0)	17 (11.3)	23 (14.7)	
	Undefined	76 (24.8)	30 (19.9)	46 (29.5)	

BMI, body mass index; NIHSS, National Institute of Health Stroke Scale; TOAST, Trial of ORG 10172 in Acute Stroke Treatment; MCA, Middle cerebral artery; ACA, Anterior cerebral artery; PCA, Posterior cerebral artery; AchA, Anterior choroidal artery.

Data are given as mean ± standard deviation (SD) for continuous variables, median with limits of the interquartile range [25^th^-75^th^ percentile] for ordinal variables and absolute (n) as well as relative (%) frequency for categorical variables. To determine significant differences between female and male patients we performed a t-test for continuous variables and a chi-squared test for categorical variables and reported the resulting p-values.

### 2.2. Input data and outcomes

This study includes a total of 43 stroke-related baseline variables in four input subdomains. They consisted of 6 demographic and 16 clinical variables, 10 serological markers and 11 MRI parameters as listed in Table 1. Procalcitonin serum levels, which have previously been identified as a prognostic marker for 30-day mortality after stroke (18), had to be excluded since this variable had more than 15% missing values. The outcomes included measures of functional recovery (mRS and BI), cognitive function (MMSE and TICS-M), depression (CES-D) and survival. The mRS and BI were assessed at patient discharge, and 1 year post-stroke. Cognitive impairment was evaluated using the MMSE at discharge and later with the TICS-M at 1 and 3 years. CES-D and survival were also assessed 1 and 3 years after the index event. The follow-up process included an initial telephone assessment of cognitive function, followed by a structured interview conducted either by phone or mail. Table 2 shows the distribution of outcomes in the dataset, their respective follow-up time points, and the cut-off points for good vs. poor clinical outcome as defined by clinical scoring gold standards.

**Table 2:** Cut-offs and distribution of outcomes, listed as frequency for patient numbers in total, males, and females.

**Distribution of outcomes in patient population**			
**Outcome**	**Time points**	**Good outcome**, ***n*** **(total/female/male)**	**Poor outcome**, ***n*** **(total/female/male)**
mRS	PD	221/110/111	86/41/45
	Year 1	193/89/104	40/27/13
BI	PD	263/125/138	44/26/18
	Year 1	195/90/10	7/6/1
MMSE	PD	271/126/145	29/21/8
TICS-M	Year 1	147/69/78	48/32/16
	Year 3	125/60/65	19/8/11
CES-D	Year 1	163/79/93	48/35/13
	Year 3	132/53/79	30/19/11
Mortality	Year 1	271/132/139	36/19/17
Year 3	142/78/64	165/73/92
**Cut-off points for good vs. poor outcome**			
**Outcome**	**Total points**	**Good outcome**	**Poor outcome**
mRS	0–6	0–2	3–6
BI	0–100	61–100	0–60
MMSE	0–30	24–30	0–23
TICS-M	0–50	30–50	0–29
CES-D	0–60	0–15	16–60

mRS, modified Rankin Scale; BI, Barthel Index; MMSE, Mini-Mental State Examination; TICS-M, Modified Telephone Interview for Cognitive Status; CES-D, Epidemiologic Studies Depression Scale; PD, patient discharge.

### 2.3. Machine learning analysis

**Figure 2:**
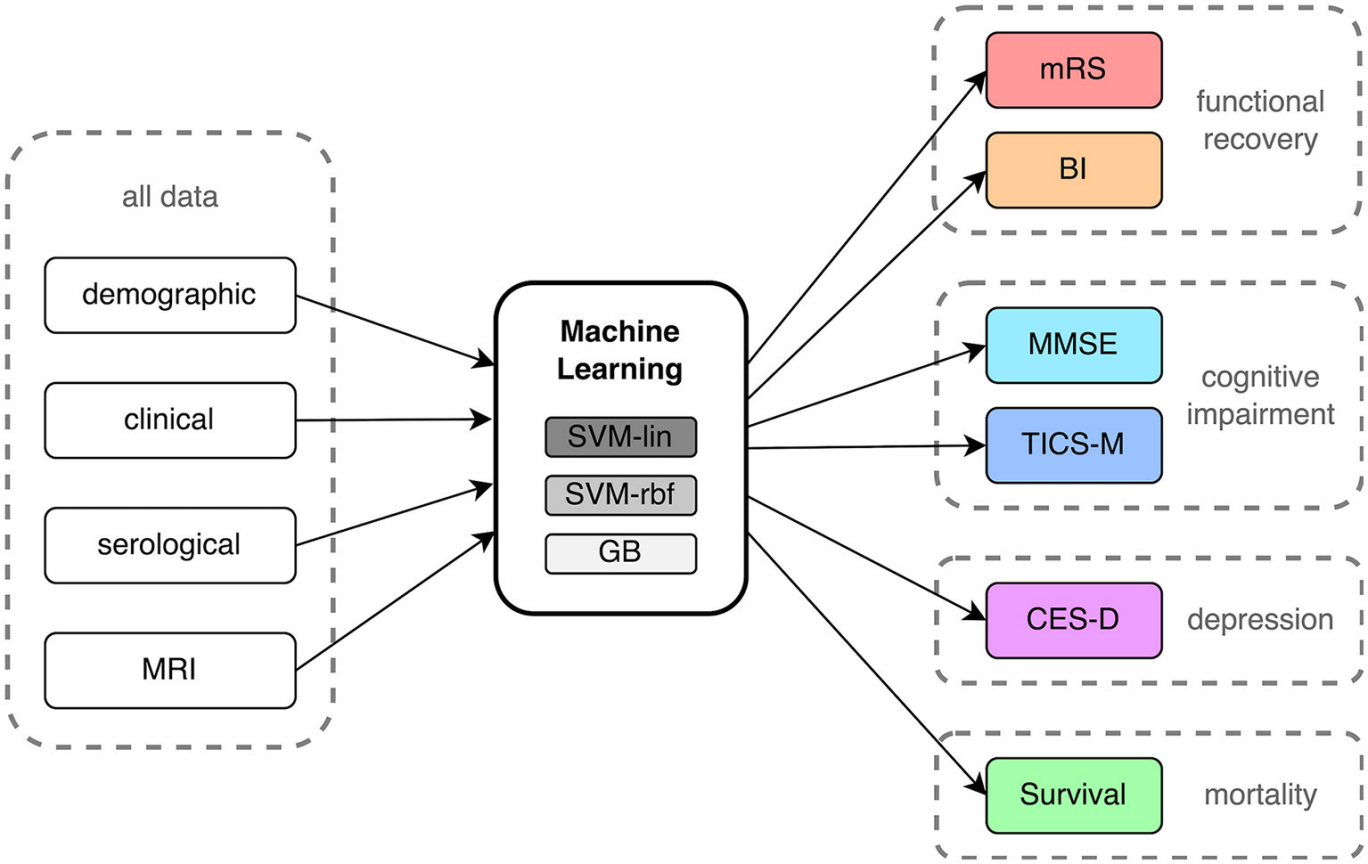
Process flow of input data, machine learning analysis and outcome prediction. mRS, modified Rankin Scale; BI, Barthel Index; MMSE, Mini-Mental State Examination; TICS-M, Modified Telephone Interview for Cognitive Status; CES-D, Epidemiologic Studies Depression Scale; SVM-lin, Support Vector Machine with linear kernel; SVM-rbf, Support Vector Machine with radial basis function kernel; GB, Gradient Bossting Classifier; MRI, Magnetic resonance imaging.

The aim of this study was to conduct a systematic comparison of ML-based outcome prediction models after first-ever ischemic stroke. To accomplish this, a linear model, a non-linear model, and a tree-based model were selected for comparison (see Figure 2). To reduce complexity and potential problems brought on by multiple comparisons, a small set of three ML algorithms were selected. A Support Vector Machine (SVM) with linear kernel (SVM-lin) (19) and a SVM with radial basis function kernel (SVM-rbf) (20) were chosen as linear and non-linear models due to their strong performance in previous studies and the ability to directly compare them (6, 16, 21). Similarly, Gradient Boosting (GB) (22) was chosen as the tree-based classifier due to its superior performance and when compared to other tree-based models (23, 24). We compensated for missing data in the training and validation set with Multiple Imputation using Chained Equations (MICE) (25). The outcome class imbalances in the training set were counteracted with the Synthetic Minority Over-sampling Technique (SMOTE) (26) and random oversampling (27). Categorical input features were transformed using one-hot encoding. Then, models were carefully evaluated using ten times repeated 5-fold nested cross-validation with fixed seed to increase robustness (28). Here the data is split into five training (80%) and test sets (20%). Each of these training sets is then subdivided into further five training (80%) and validation sets (20%). The hyperparameters of the ML models (listed in Supplementary Table S1) have been optimized on these training and validation sets *via* grid search before finally being evaluated on the unseen data of the test sets.

Performance of each model was evaluated using balanced accuracy (BA), area under the receiver operating characteristic curve, sensitivity, specificity, likelihood ratio (LR) and Integrated Discrimination Improvement index (IDI). BA is the arithmetic mean of sensitivity and specificity while the receiver operating characteristics curve (ROC) plots the true positive rate in relation to the false positive rate of the ML models. The area under the curve (AUC) of the ROC is routinely used as a measure of performance in ML. For each outcome, we reported the mean BA and AUC along with their standard deviation (SD) for ten iterations of 5-fold nested cross-validation. The LR compares the fit of two models by taking the ratio of their likelihoods (29) while the IDI ranks the model according to the change of the discrimination slopes (30). To test for statistical significance, we performed non-parametric permutation testing (31). Here, the exact same ML analysis and nested cross-validation procedure was performed a hundred times on randomly permuted ground truth labels before being compared to the original results. Results were considered statistically significant below *p* ≤ 0.05 and *p* ≤ 0.01 after Bonferroni correction for multiple comparisons (3 ML algorithms × 5 feature subsets). We used the Python 3.6 programming language with the scikit-learn, pandas, statsmodel, matplotlib and seaborn packages for all analyses and visualizations.

### 2.4. Feature importance and Shapley values

In order to discern feature importance we implemented Shapley values using the SHAP (SHapley Additive exPlanations) framework (32). This statistic is a solution concept originating from cooperative game theory which calculates the relative importance of an input feature for the final prediction result and has already demonstrated convincing results in biomedical and clinical research applications (33, 34). Shapley values are calculated by determining the average marginal contribution of each feature over all possible combinations of input features. This is done by analyzing the effect of each feature on the prediction when it is included or excluded, while also taking into account the dependencies between features. For the purposes of this study, we implemented the Kernel SHAPexplainer which acts as a specially-weighted local linear regression (32).

## 3. Results

Out of the 621 PROSCIS-B patients 125 had no MRI associated with their study ID and in 5 further cases we were unable to locate the MRI data. This resulted in 491 patients with imaging data out of which 255 had received a 3T scan at the Center of Stroke Research Berlin (CSB) and 236 had been processed on scanners at Charité - Universitätsmedizin Berlin ranging from 1 to 1.5T, all of which were Siemens MRI units. In 56 cases the imaging data could not be delineated due to missing sequences or motion artifacts and in 8 cases participants had retracted their consent for the study which resulted in a total of 427 fully delineated cases. The final balanced dataset consisted of 307 patients. There was a loss to follow-up of 74 patients (24.1%) in mRS, 105 patients (34.2%) in BI, 51 patients (26.2%) in TICS-M, and 49 patients (23.2%) in CES-D from the initial sample size. No loss was observed for mortality.

**Figure 3:**
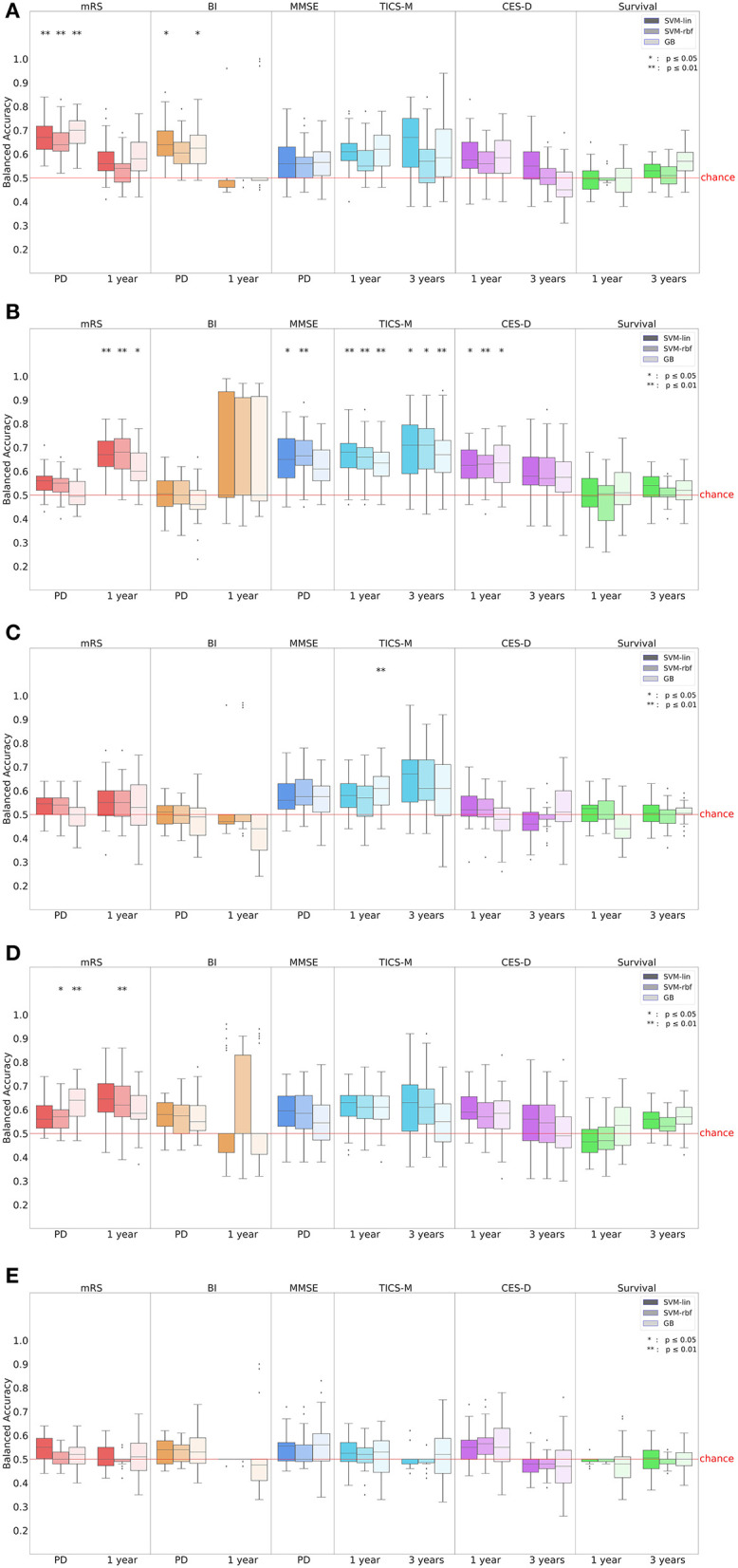
Prediction performance in balanced accuracy (BA) for all outcomes, time points and input subdomains. In **(A)** all input parameters were considered while **(B–E)** show the results of the **(B)** demographic, **(C)** clinical, **(D)** serological and **(E)** MRI input subdomain. Results for BI after 1 year were unreliable due to the extreme class imbalance in the dataset (see Table 2). mRS, modified Rankin Scale; BI, Barthel Index; MMSE, Mini-Mental State Examination; TICS-M, Modified Telephone Interview for Cognitive Status; CES-D, Epidemiologic Studies Depression Scale; SVM-lin, Support Vector Machine with linear kernel; SVM-rbf, Support Vector Machine with radial basis function kernel; GB, Gradient Bossting Classifier; MRI, Magnetic resonance imaging.

We evaluated and ranked the performance of the ML models using the metrics of BA and AUC. The results of these analyses can be found in Supplementary Tables S2–S6. In Figure 3, we show the performance in BA for all outcomes (mRS, BI, MMSE, TICS-M, CES-D, and survival), time points, and ML models (SVM-lin, SVM-rbf and GB). Additionally, we calculated the Integrated IDI and LR to provide further insight into the models' performance. The detailed results are reported in Supplementary Tables S7–S11. While the LR revealed no significant differences between the ML models it is important to note that the results obtained from the BA, AUC and the LR should be viewed independently, as they are based on different methods of evaluating the models' performance. Although in many cases the performance of the three ML models was at a comparable level the strongest predictive performance overall was achieved by SVM-rbf for TICS-M after 3 years (BA ± SD = 0.7 ± 0.13; AUC ± SD = 0.76 ± 0.13; p ≤ 0.05) using the demographic input subdomain. Table 3 states the most important predictors according to the Shapley values. The following paragraphs will list significant results (*p* ≤ 0.05 or *p* ≤ 0.01 Bonferroni corrected) according to the permutation test for each outcome per input subdomain.

**Table 3:** Best prediction results and most important predictors for all outcomes as determined *via* Shapley values.

**Outcome**	**Time**	**Model**	**Input**	**Mean absolute SHAP value**	**Variables**
mRS	PD	GB	All	0.68 [0.64, 0.72]	NIHSS
				0.44 [0.41, 0.47]	hsCRP
				0.21 [0.19, 0.24]	Glucose
				0.18 [0.15, 0.21]	Cholesterol
				0.18 [0.15, 0.20]	Supra-/Infratentorial
	Year 1	SVM-rbf	Demographic	0.52 [0.47, 0.57]	Waist circumference [cm]
				0.50 [0.46, 0.54]	Sex
				0.47 [0.43, 0.51]	Age
				0.37 [0.33, 0.41]	Education [years]
				0.19 [0.15, 0.22]	BMI
BI	PD	SVM-lin	All	1.11 [1.05, 1.18]	NIHSS
				0.61 [0.57, 0.65]	Smoking
				0.46 [0.42, 0.49]	TOAST classification
				0.41 [0.36, 0.45]	Infarct pattern
				0.37 [0.34, 0.41]	Infarct origin
TICS-M	Year 1	SVM-lin	Demographic	0.68 [0.62, 0.73]	Education
				0.51 [0.46, 0.56]	Age
				0.40 [0.34, 0.46]	BMI
				0.20 [0.17, 0.23]	Sex
				0.19 [0.16, 0.21]	Hip circumference [cm]
	Year 3	SVM-rbf	Demographic	1.32 [1.16, 1.49]	Education [years]
				0.54 [0.48, 0.60]	Age
				0.42 [0.36, 0.47]	Sex
				0.38 [0.31, 0.44]	Waist circumference [cm]
				0.36 [0.31, 0.42]	Hip circumference [cm]
MMSE	PD	SVM-rbf	Demographic	0.48 [0.43, 0.53]	Education [years]
				0.36 [0.33, 0.38]	Sex
				0.35 [0.30, 0.41]	Age
				0.14 [0.11, 0.17]	Waist circumference [cm]
				0.13 [0.10, 0.15]	BMI
CES-D	Year 1	GB	Demographic	0.52 [0.49, 0.55]	Education [years]
				0.42 [0.38, 0.46]	Sex
				0.36 [0.31, 0.41]	BMI
				0.29 [0.25, 0.32]	Hip circumference [cm]
				0.21 [0.17, 0.26]	Waist circumference [cm]

mRS, modified Rankin Scale; BI, Barthel Index; MMSE, Mini-Mental State Examination; TICS-M, Modified Telephone Interview for Cognitive Status; CES-D, Epidemiologic Studies Depression Scale; PD, patient discharge; SVM-lin, Support Vector Machine with linear kernel; SVM-rbf, Support Vector Machine with radial basis function kernel; GB, Gradient Boosting Classifier; BMI, body mass index; NIHSS, National Institute of Health Stroke Scale; TOAST, Trial of ORG 10172 in Acute Stroke Treatment.

The mean absolute SHAP value is reported with 95% confidence interval.

### 3.1. Modified Rankin Scale

The highest prediction score for mRS at patient discharge was achieved by GB (BA ± SD = 0.69 ± 0.07; AUC ± SD = 0.77 ± 0.06; *p* ≤ 0.01) followed by SVM-lin (BA ± SD = 0.67 ± 0.07; AUC ± SD = 0.74 ± 0.07; *p* ≤ 0.01) and SVM-rfb (BA ± SD = 0.65 ± 0.06; AUC ± SD = 0.77 ± 0.06; *p* ≤ 0.01) using all input parameters. In the serological input subdomain GB (BA ± SD = 0.63 ± 0.07; AUC ± SD = 0.68 ± 0.08; *p* ≤ 0.01) and SVM-rbf (BA ± SD = 0.57 ± 0.06; AUC ± SD = 0.63 ± 0.07; *p* ≤ 0.05) attained significant prediction results. The top five predictors using all input parameters were National Institutes of Health Stroke Scale (NIHSS), hsCRP, glucose, cholesterol and supra-/infratentorial infarct location.

The mRS after 1 year could best be predicted using the demographic input subdomain by SVM-rbf (BA ± SD = 0.68 ± 0.09; AUC ± SD = 0.73 ± 0.01; *p* ≤ 0.01) followed by SVM-lin (BA ± SD = 0.67 ± 0.08; AUC ± SD = 0.73 ± 0.01; *p* ≤ 0.01) and GB (BA ± SD = 0.61 ± 0.08; AUC ± SD = 0.66 ± 0.09; *p* ≤ 0.05). In the serological input subdomain, SVM-rbf (BA ± SD = 0.63 ± 0.1; AUC ± SD = 0.64 ± 0.12; *p* ≤ 0.01) led in prediction results. Waist circumference, sex, age, education, and BMI were the leading predictors in the demographic input subdomain.

### 3.2. Barthel Index

For BI at patient discharge, SVM-lin (BA ± SD = 0.65 ± 0.08; AUC ± SD = 0.73 ± 0.11; *p* ≤ 0.05) and GB (BA ± SD = 0.63 ± 0.08; AUC ± SD = 0.74 ± 0.07; *p* ≤ 0.05) achieved significant prediction results using all input parameters. The strongest predictors were NIHSS, smoking, the Trial of ORG 10172 in Acute Stroke Treatment (TOAST) classification, infarct pattern and infarct origin. However, BI after 1 year could not be predicted by any model.

### 3.3. Mini-Mental State Examination

The leading ML models for predicting MMSE at patient discharge were SVM-rbf (BA ± SD = 0.67 ± 0.09; AUC ± SD = 0.71 ± 0.11; *p* ≤ 0.01) and SVM-lin (BA ± SD = 0.65 ± 0.1; AUC ± SD = 0.7 ± 0.1; *p* ≤ 0.05) using the demographic input subdomain with education, sex, age, waist circumference and BMI being the most important predictors.

### 3.4. Modified Telephone Interview for Cognitive Status

The best predictions for TICS-M after 1 year were by SVM-lin (BA ± SD = 0.67 ± 0.09; AUC ± SD = 0.73 ± 0.09; *p* ≤ 0.01), SVM-rbf (BA ± SD = 0.65 ± 0.09; AUC ± SD = 0.72 ± 0.09; *p* ≤ 0.01) and GB (BA ± SD = 0.63 ± 0.08; AUC ± SD = 0.69 ± 0.11; *p* ≤ 0.01) using the demographic input subdomain. Further significant prediction results were achieved by GB (BA ± SD = 0.6 ± 0.08; AUC ± SD = 0.66 ± 0.1; *p* ≤ 0.01) using the clinical input subdomain. The top five predictors in the demographic input subdomain were education, age, BMI, sex, and hip circumference. TICS-M after 3 years was most successfully predicted by SVM-rbf (BA ± SD = 0.7 ± 0.13; AUC ± SD = 0.76 ± 0.13; *p* ≤ 0.05), SVM-lin (BA ± SD = 0.69 ± 0.14; AUC ± SD = 0.77 ± 0.13; *p* ≤ 0.05) and GB (BA ± SD = 0.68 ± 0.12; AUC ± SD = 0.74 ± 0.13; *p* ≤ 0.01) using the demographic input subdomain. Education, age, sex, waist circumference, and hip circumference were the leading variables.

### 3.5. Center for epidemiologic studies depression scale

For the prediction of CES-D after 1 year the use of the demographic input subdomain led to a significant prediction performance by GB (BA ± SD = 0.63 ± 0.09; AUC ± SD = 0.7 ± 0.1; *p* ≤ 0.05), SVM-lin (BA ± SD = 0.63 ± 0.08; AUC ± SD = 0.68 ± 0.1; *p* ≤ 0.05) and SVM-rbf (BA ± SD = 0.62 ± 0.07; AUC ± SD = 0.7 ± 0.09; *p* ≤ 0.01). The strongest predictors were education, sex, BMI as well as hip and waist circumference. No ML model achieved significant prediction results for CES-D after 3 years.

### 3.6. Survival

Survival within 1 or 3 years could not be predicted reliably by any model.

## 4. Discussion

To the best of our knowledge, this is the first study to apply highly comparable standardized ML models to predict a wide range of long-term patient outcomes including functional recovery, cognitive impairment, depression, and mortality from a single, homogenous patient collective. While functional recovery scores like mRS and BI are often used as primary outcome endpoints in most major stroke cohorts, cognitive impairment and depression play a vital role in terms of long-term patient outcome. Up to 80% of patients are affected by cognitive impairment post-stroke and up to 30% will develop a clinically relevant depression within 2 years after the index event (35, 36). These factors not only negatively affect functional recovery by decreasing a patient's capability for actively participating in rehabilitation measures but also disrupt their social integration. Although numerous previous studies have used similar ML models to predict functional recovery after stroke (5), here we demonstrate the accuracy of ML models to predict post-stroke cognitive status and depression up to 3 years post-stroke, as well as functional recovery.

**Figure 4:**
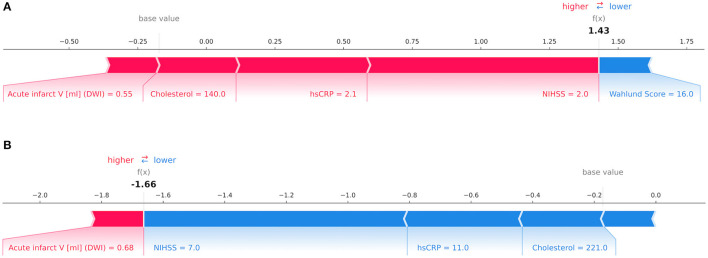
Decision-making process by the Gradient Boosting Classifier for the modified Rankin Scale (mRS) at patient discharge on the level of individual patients depicted *via* Shapley values. The relative importance of an input variable can be quantified by its Shapley value and represented by the length of a bar. In this example, features in red counted toward a good outcome while blue features signified poor outcome for mRS at patient discharge. In **(A)** a patient with a mRS score of 1 point was correctly classified as having a good outcome with variables such as low National Institutes of Health Stroke Scale (NIHSS), high-sensitivity C-reactive protein (hsCRP), cholesterol and acute infarct volume in Diffusion-weighted imaging (DWI) outweighing a high Wahlund Score. In **(B)** a patient with a mRS score of 4 points was correctly predicted as having poor outcome due to high NIHSS, hsCRP and cholesterol whilst offsetting a low acute infarct volume in DWI. In both instances the decision was made by considering the total impact of all features.

Our results are in line with previous studies in identifying NIHSS as the leading predictor for mRS at patient discharge amongst all input variables (37, 38). Increased levels of hsCRP were correlated with poor clinical outcome which supports findings reported by den Hertog et al. (39) in acute stroke. Interestingly, waist circumference was the leading predictor for mRS after 1 year. Being underweight (BMI < 18.5 kg/m^2^) has previously been associated with unfavorable outcomes in terms of mortality and functional recovery in previous studies (40). Figure 4 illustrates the decision-making process of GB for mRS at patient discharge on a single-subject level.

In a study by Monteiro et al. (6) various ML models were applied to predict mRS after 3 months from 425 patients using 152 input variables. The best performance using baseline variables was achieved using a Random Forest (RF) classifier with an AUC of 0.808 ± 0.085. In a separate study by Heo et al. (7) a DNN was used on 3,522 patients and achieved a classification accuracy of AUC = 0.888 with no reported SD. However, the authors did not mention whether cross-validation or repetition were used, which are important for developing a robust ML model and avoiding over-fitting. In a study by Li et al. (21) predicting mRS after 6 months a SVM (AUC = 0.865; 95% CI 0.823–0.907) performed comparably well with six other models, including a RF classifier (AUC = 0.874; 95% CI 0.835–0.912) and a DNN (AUC 0.867; 95% CI 0.827–0.908). In contrast, in our study, for mRS at patient discharge the SVM-lin (AUC ± SD = 0.74 ± 0.07) was outperformed by GB (AUC ± SD = 0.77 ± 0.06). However, comparing the results of these studies is challenging due to variations in follow-up time points, input variables, methodology, and performance measures. Nevertheless, it appears that SVMs tend to perform similarly to, or worse than, tree-based classifiers or DNNs for predicting mRS outcomes.

Considerable overlap exists between mRS and BI in the development of functional recovery post stroke (41). This is reflected in NIHSS being the leading predictor for BI at patient discharge. Our results also confirm the relative importance of stroke origin for this outcome (42). The BI after one year could not be predicted—this may be due to the extreme class imbalance of this outcome (see Table 2). In contrast, in a study by den Hertog et al. (39) a ML model for identifying prognostic factors for motor and cognitive improvement after post-stroke rehabilitative training was developed based on a SVM-lin. The model included 55 patients and the results of the ischemic test set reported performance scores of correlation = 0.75, MADP = 87,03% and RMSE = 21,74 for BI. The most important parameters for the prediction were identified as the Functional Independence Measure and BI at patient discharge as well as serological markers such as Platelet-to-lymphocyte ratio, Red Cell Distribution Width and Lymphocytes.

Amongst the leading predictors for cognitive function post-stroke were demographic factors such as education, age and BMI which confirms previously published results (43, 44). While our findings are in line with the results by Casanova et al. (45) and Aschwanden et al. (46) their studies additionally identified the importance of socioeconomic status and ethnicity in terms of cognitive function post-stroke. Unfortunately, in the current study, these variables could not be accounted for.

Education being the top predictor for levels of depression after 1 year is in accordance with several studies linking low education level to an increased risk of post-stroke depression (47). Previous studies have found a significant association between higher waist circumference with an elevated rate of depression (48). In the current analysis, female sex was also identified as an important predictor of depression (49). A study by Hama et al. (50) achieved an impressive AUC above 0.90 for the prediction of post-stroke depression using a probabilistic artificial neural network on 274 stroke inpatients at the Hibino Hospital. The predicted clinical score was the Hospital Anxiety and Depression Scale and its lead predictors were the Japanese Perceived Stress Scale, the Symbol Digit Modalities Test, tapping span backward, visual cancellation Kana time and the Continuous Performance Test. This jump in prediction accuracy may be explained in part by the inclusion of these very specific test scores.

### 4.1. Methodological considerations

While many previous ML-based studies achieved noteworthy results, there are some potentially problematic methodological factors to consider: ideally, a ML model is trained and tested on numerous different samples in order to create a robust predictor for new, unseen data (51). In face of limited clinical data, it is crucial to include a re-sampling procedure to ensure effective training (52). Additionally, few studies performed more than one iteration of their analyses which negatively impacts robustness (28). In our study, we accounted for these factors by using a repeated 5-fold nested cross-validation. Furthermore, many studies use datasets and ML methods specific to the purpose of predicting an individual outcome. This impedes comparability as it remains unclear whether differences in performance are based on variations in input data or technical aspects of the ML analysis (5). Neglecting to balance these datasets regarding age and sex may also lead to biased results (53). We therefore balanced the dataset according to age and sex and predicted a range of clinical outcomes from the same dataset using three classical ML models while ensuring independence between training and test data. In addition, and in contrast to previous ML studies, we estimated the relative importance of features using Shapley values allowing to assess the impact of different input features for clinical outcome prediction in individual patients (see Figure 4).

### 4.2. Clinical implications

In the coming years, the advancement of big data analytics based on collaboration networks and electronic health records is set to drive a paradigm shift in clinical research (54). Novel automated and computer-based methods will play a key role in making use of increasing datasets and processing power. Therefore, we take a crucial step forward in the application of ML-based research methods to one of the most common and severe diseases around the globe and show that established as well as less traditional risk predictors can be identified and reproduced with ML techniques even in a limited sample size.

There is currently no established prediction score for depression outcomes following ischemic stroke. However, there are already a variety of scores available in the scientific literature for predicting functional outcomes (such as the Wang et al. (55) and ASTRAL (56) scores), cognitive outcomes (such as the CHANGE (57) and SIGNAL2 (58) scores), and mortality outcomes (such as the iScore (59) and PLAN (60) scores). In future studies, the aim should be to develop a universal model that can predict multiple outcomes-including functional recovery, cognitive impairment, depression, and mortality outcomes-using a basic set of variables such as NIHSS, education, sex, age, or BMI. This model would ideally be an easy-to-use tool for clinicians in real-world medical practice and act as an AI-based clinical decision support system (CDSS). The implementation of CDSS has been shown to be a cost-effective and efficient method for enhancing clinical workflow and decision-making (61). CDSSs have the potential to enhance patient safety by mitigating the occurrence of oversights and treatment errors. In the case of stroke, functional recovery is heavily dependent on rehabilitation measures which in turn requires adequate cognitive function and management of post-stroke depression (62, 63). The ability of CDSSs to alert providers to potential challenges in the management process can provide valuable guidance for more personalized rehabilitation programs and patient-tailored secondary prevention strategies, ultimately improving post-stroke outcomes.

### 4.3. Limitations

This study has several limitations that warrant discussion. First and foremost, this study had a limited sample size, the outcome classes were imbalanced, and an external control dataset was lacking. The application of 5-fold nested cross-validation, SMOTE and random oversampling partially counteract these limitations. To avoid shortcut learning and develop a model representative of the general population, we balanced our dataset by age and sex. Shortcut learning occurs when the model relies heavily on easily observable features like age rather than underlying causes, leading to potential biases and inaccuracies when applied to individuals outside the trained age range. However, this approach does not account for the natural incidence variation within the population, which may impact the ML model's predictions. Additionally, most of the patients included in this study had relatively mild to moderate strokes (NIHSS median of 2 (1–4)); this may have negatively affected prediction performance and limits generalizability to more severely affected stroke cohorts. There was also no data available on whether patients entered a rehabilitation program post-stroke, or which secondary prevention strategies were initiated. Therefore, these factors could not be accounted for in terms of post-stroke outcome endpoints in this analysis.

## 5. Conclusion

Based on a systematic comparison, the results of this study demonstrated the viability of ML-based outcome prediction after first-ever ischemic stroke for functional recovery, cognitive function, depression, and mortality. Compared to group-based statistical analyses, the advantage of ML-techniques is their ability to make predictions on a single-subject level by considering a multitude of variables which is key for future application in clinical routine. Furthermore, we extracted the most important prognostic variables for each outcome. On the one hand, the results confirmed several already established prognostic markers and on the other identified novel candidates such as education, hsCRP and waist circumference as relevant predictors of important clinical endpoints. However, further studies are needed to confirm these findings and to establish their clinical viability.

## Data availability statement

The PROCIS-B data is available upon request from TL. The code and results data are available upon request from KR.

## Ethics statement

The studies involving human participants were reviewed and approved by the Ethics Committee of the Charité - Universitätsmedizin Berlin (EA1/218/09). The patients/participants or their legal representative provided their written informed consent to participate in this study.

## Author contributions

LF, KV, AK, and KR: conceptualization. LF, UT, KV, AK, HA, SP, and KR: data curation. LF, UT, and KR: formal analysis, methodology, visualization, and software. LF, TL, and KR: project administration. LF: writing–original draft. KV, AK, ES, SH, SP, PS, TL, ME, and KR: writing–review and editing. KV, TL, and KR: resources. KV and KR: supervision. All authors contributed to the article and approved the submitted version.
