# Supplementary material for: Machine learning-based prediction of clinical outcomes after first-ever ischemic stroke
Source: Front Neurol. 2023 Feb 21;14:1114360. doi: 10.3389/fneur.2023.1114360 (PMC9990416; doi:10.3389/fneur.2023.1114360)
Supplement: Supplementary file 1 [file Table_1.docx]

SUPPLEMENTARY

Machine learning-based prediction of clinical outcomes
after first-ever ischemic stroke

Lea Fast, Uchralt Temuulen, Kersten Villringer, Anna Kufner, Huma Fatima Ali, Eberhard Siebert, Shufan Huo, Sophie K. Piper, Pia Sophie Sperber, Thomas Liman, Matthias Endres, Kerstin Ritter

Table S1. Hyperparameters of the machine learning models.

| **Model** | **Hyperparameter** | **Values** |
| --- | --- | --- |
| SVM-lin | k | all |
|  | C | 1, 10, 100, 1000 |
| SVM-rbf | k | all |
|  | Kernel | rbf |
|  | C | 1, 10, 100, 1000 |
|  | Gamma | 0.001, 0.0001 |
| GB | max_depth | 8, 16, 32, 40, 50 |
|  | max_features | sqrt, auto, log2 |
|  | n_estimators | 25, 50, 75, 100 |
|  | min_samples_split | 0.1, 0.3 |
|  | min_samples_leaf | 0.3, 0.5 |

Abbreviations: SVM-lin, Support Vector Machine with linear kernel; SVM-rbf, Support Vector Machine with radial basis function kernel; GB, Gradient Boosting Classifier

Table S2. Results for all input variables and all models listed in BA±SD, AUC±SD, Sensitivity±SD, Specificity±SD and p-values according to the permutation test.

| **Labels** | **Time** | **Model** | **BA±SD** | **AUC±SD** | **Sensitivity±SD** | **Specificity±SD** | **P-value** |
| --- | --- | --- | --- | --- | --- | --- | --- |
| mRS | PD | SVM-lin | 0.67±0.07 | 0.74±0.07 | 0.85±0.06 | 0.49±0.13 | 0 |
|  |  | SVM-rbf | 0.65±0.06 | 0.77±0.06 | 0.91±0.06 | 0.4±0.14 | 0 |
|  |  | GB | 0.69±0.07 | 0.77±0.06 | 0.8±0.06 | 0.57±0.13 | 0 |
|  | 1 year | SVM-lin | 0.58±0.08 | 0.66±0.09 | 0.85±0.07 | 0.3±0.15 | 1 |
|  |  | SVM-rbf | 0.53±0.06 | 0.68±0.09 | 0.95±0.04 | 0.11±0.11 | 1 |
|  |  | GB | 0.59±0.09 | 0.7±0.09 | 0.8±0.07 | 0.39±0.18 | 0,45 |
| BI | PD | SVM-lin | 0.65±0.08 | 0.73±0.11 | 0.91±0.03 | 0.39±0.17 | 0,03 |
|  |  | SVM-rbf | 0.6±0.07 | 0.73±0.1 | 0.97±0.03 | 0.23±0.15 | 0,18 |
|  |  | GB | 0.63±0.08 | 0.74±0.07 | 0.83±0.06 | 0.42±0.16 | 0,03 |
|  | 1 year | SVM-lin | 0.51±0.12 | 0.68±0.17 | 0.95±0.03 | 0.06±0.24 | 1 |
|  |  | SVM-rbf | 0.5±0.01 | 0.58±0.19 | 1.0±0.01 | 0.0±0.0 | 1 |
|  |  | GB | 0.55±0.16 | 0.64±0.25 | 0.98±0.03 | 0.12±0.33 | 1 |
| TICS-M | 1 year | SVM-lin | 0.61±0.08 | 0.69±0.08 | 0.8±0.07 | 0.41±0.16 | 0,225 |
|  |  | SVM-rbf | 0.57±0.07 | 0.68±0.09 | 0.9±0.08 | 0.23±0.15 | 0,96 |
|  |  | GB | 0.61±0.08 | 0.69±0.1 | 0.79±0.1 | 0.44±0.18 | 0,09 |
|  | 3 years | SVM-lin | 0.65±0.13 | 0.77±0.11 | 0.86±0.06 | 0.43±0.26 | 0,54 |
|  |  | SVM-rbf | 0.57±0.11 | 0.75±0.11 | 0.93±0.06 | 0.22±0.21 | 1 |
|  |  | GB | 0.62±0.14 | 0.74±0.14 | 0.9±0.06 | 0.34±0.29 | 0,75 |
| MMSE | PD | SVM-lin | 0.57±0.09 | 0.71±0.1 | 0.91±0.04 | 0.23±0.19 | 1 |
|  |  | SVM-rbf | 0.56±0.07 | 0.71±0.09 | 0.96±0.03 | 0.16±0.14 | 1 |
|  |  | GB | 0.56±0.08 | 0.66±0.1 | 0.85±0.04 | 0.27±0.18 | 1 |
| CES-D | 1 year | SVM-lin | 0.58±0.09 | 0.62±0.1 | 0.8±0.08 | 0.37±0.19 | 0,915 |
|  |  | SVM-rbf | 0.57±0.07 | 0.64±0.11 | 0.93±0.06 | 0.2±0.14 | 1 |
|  |  | GB | 0.59±0.09 | 0.66±0.1 | 0.78±0.07 | 0.4±0.18 | 0,195 |
|  | 3 years | SVM-lin | 0.55±0.08 | 0.56±0.1 | 0.78±0.1 | 0.31±0.17 | 1 |
|  |  | SVM-rbf | 0.5±0.06 | 0.53±0.11 | 0.93±0.06 | 0.08±0.1 | 1 |
|  |  | GB | 0.47±0.08 | 0.45±0.13 | 0.77±0.09 | 0.17±0.16 | 1 |
| Survival | 1 year | SVM-lin | 0.5±0.06 | 0.53±0.11 | 0.89±0.05 | 0.11±0.11 | 1 |
|  |  | SVM-rbf | 0.5±0.02 | 0.55±0.1 | 0.99±0.01 | 0.01±0.03 | 1 |
|  |  | GB | 0.5±0.07 | 0.48±0.12 | 0.81±0.05 | 0.18±0.13 | 1 |
|  | 3 years | SVM-lin | 0.53±0.04 | 0.55±0.05 | 0.5±0.08 | 0.56±0.07 | 1 |
|  |  | SVM-rbf | 0.51±0.05 | 0.5±0.07 | 0.35±0.15 | 0.68±0.14 | 1 |
|  |  | GB | 0.56±0.06 | 0.61±0.05 | 0.48±0.15 | 0.65±0.1 | 0,09 |

Abbreviations: mRS, Modified Rankin Scale; BI, Barthel Index; MMSE, Mini-Mental State Examination; TICS-M, Modified Telephone Interview for Cognitive Status; CES-D, Epidemiologic Studies Depression Scale; SVM-lin, Support Vector Machine with linear kernel; SVM-rbf, Support Vector Machine with radial basis function kernel; GB, Gradient Boosting Classifier; PD, Patient discharge; BA, Balance accuracy; AUC, Area under the curve; SD, Standard deviation

Table S3. Results for the demographic input subdomain and all models listed in BA±SD, AUC±SD, Sensitivity±SD, Specificity±SD and p-values according to the permutation test.

| **Labels** | **Time** | **Model** | **BA±SD** | **AUC±SD** | **Sensitivity±SD** | **Specificity±SD** | **P-value** |
| --- | --- | --- | --- | --- | --- | --- | --- |
| mRS | PD | SVM-lin | 0.56±0.05 | 0.57±0.07 | 0.64±0.08 | 0.48±0.1 | 0,675 |
|  |  | SVM-rbf | 0.54±0.05 | 0.57±0.07 | 0.6±0.09 | 0.49±0.1 | 0,33 |
|  |  | GB | 0.51±0.06 | 0.54±0.07 | 0.65±0.09 | 0.36±0.1 | 1 |
|  | 1 year | SVM-lin | 0.67±0.08 | 0.73±0.1 | 0.7±0.08 | 0.65±0.17 | 0 |
|  |  | SVM-rbf | 0.68±0.09 | 0.73±0.1 | 0.7±0.08 | 0.65±0.19 | 0 |
|  |  | GB | 0.61±0.08 | 0.66±0.09 | 0.72±0.09 | 0.5±0.19 | 0,03 |
| BI | PD | SVM-lin | 0.51±0.07 | 0.49±0.1 | 0.65±0.06 | 0.36±0.13 | 1 |
|  |  | SVM-rbf | 0.5±0.08 | 0.48±0.09 | 0.61±0.15 | 0.4±0.19 | 1 |
|  |  | GB | 0.47±0.07 | 0.45±0.09 | 0.68±0.08 | 0.27±0.11 | 1 |
|  | 1 year | SVM-lin | 0.66±0.23 | 0.86±0.15 | 0.92±0.07 | 0.39±0.49 | 1 |
|  |  | SVM-rbf | 0.63±0.22 | 0.83±0.19 | 0.91±0.09 | 0.36±0.48 | 1 |
|  |  | GB | 0.63±0.22 | 0.78±0.21 | 0.92±0.08 | 0.34±0.48 | 1 |
| TICS-M | 1 year | SVM-lin | 0.67±0.09 | 0.73±0.09 | 0.69±0.06 | 0.65±0.15 | 0 |
|  |  | SVM-rbf | 0.65±0.09 | 0.72±0.09 | 0.66±0.06 | 0.65±0.17 | 0 |
|  |  | GB | 0.63±0.08 | 0.69±0.11 | 0.71±0.08 | 0.56±0.17 | 0 |
|  | 3 years | SVM-lin | 0.69±0.14 | 0.77±0.13 | 0.76±0.08 | 0.63±0.27 | 0,015 |
|  |  | SVM-rbf | 0.7±0.13 | 0.76±0.13 | 0.73±0.08 | 0.66±0.25 | 0,015 |
|  |  | GB | 0.68±0.12 | 0.74±0.13 | 0.8±0.08 | 0.56±0.23 | 0 |
| MMSE | PD | SVM-lin | 0.65±0.1 | 0.7±0.1 | 0.7±0.06 | 0.6±0.2 | 0,03 |
|  |  | SVM-rbf | 0.67±0.09 | 0.71±0.11 | 0.68±0.07 | 0.66±0.2 | 0 |
|  |  | GB | 0.62±0.09 | 0.67±0.1 | 0.75±0.06 | 0.5±0.19 | 0,105 |
| CES-D | 1 year | SVM-lin | 0.63±0.08 | 0.68±0.1 | 0.71±0.08 | 0.56±0.15 | 0,03 |
|  |  | SVM-rbf | 0.62±0.07 | 0.7±0.09 | 0.66±0.12 | 0.58±0.15 | 0 |
|  |  | GB | 0.63±0.09 | 0.7±0.1 | 0.73±0.08 | 0.53±0.17 | 0,015 |
|  | 3 years | SVM-lin | 0.6±0.1 | 0.6±0.11 | 0.7±0.1 | 0.5±0.19 | 0,555 |
|  |  | SVM-rbf | 0.59±0.1 | 0.62±0.12 | 0.66±0.11 | 0.52±0.19 | 0,195 |
|  |  | GB | 0.58±0.1 | 0.62±0.11 | 0.71±0.11 | 0.45±0.18 | 0,81 |
| Survival | 1 year | SVM-lin | 0.5±0.09 | 0.49±0.11 | 0.63±0.06 | 0.37±0.19 | 1 |
|  |  | SVM-rbf | 0.48±0.09 | 0.47±0.1 | 0.62±0.09 | 0.34±0.19 | 1 |
|  |  | GB | 0.53±0.1 | 0.53±0.11 | 0.67±0.08 | 0.39±0.18 | 1 |
|  | 3 years | SVM-lin | 0.53±0.06 | 0.55±0.07 | 0.37±0.07 | 0.7±0.1 | 1 |
|  |  | SVM-rbf | 0.5±0.04 | 0.48±0.08 | 0.32±0.22 | 0.69±0.22 | 1 |
|  |  | GB | 0.52±0.06 | 0.56±0.07 | 0.37±0.11 | 0.68±0.12 | 1 |

Abbreviations: mRS, Modified Rankin Scale; BI, Barthel Index; MMSE, Mini-Mental State Examination; TICS-M, Modified Telephone Interview for Cognitive Status; CES-D, Epidemiologic Studies Depression Scale; SVM-lin, Support Vector Machine with linear kernel; SVM-rbf, Support Vector Machine with radial basis function kernel; GB, Gradient Boosting Classifier; PD, Patient discharge; BA, Balance accuracy; AUC, Area under the curve; SD, Standard deviation

Table S4. Results for the clinical input subdomain and all models listed in BA±SD, AUC±SD, Sensitivity±SD, Specificity±SD and p-values according to the permutation test.

| **Labels** | **Time** | **Model** | **BA±SD** | **AUC±SD** | **Sensitivity±SD** | **Specificity±SD** | **P-value** |
| --- | --- | --- | --- | --- | --- | --- | --- |
| mRS | PD | SVM-lin | 0.54±0.05 | 0.56±0.07 | 0.87±0.07 | 0.21±0.08 | 1 |
|  |  | SVM-rbf | 0.53±0.05 | 0.57±0.07 | 0.91±0.07 | 0.15±0.08 | 1 |
|  |  | GB | 0.49±0.07 | 0.49±0.08 | 0.64±0.09 | 0.34±0.13 | 1 |
|  | 1 year | SVM-lin | 0.55±0.08 | 0.68±0.09 | 0.83±0.08 | 0.27±0.16 | 1 |
|  |  | SVM-rbf | 0.55±0.08 | 0.67±0.09 | 0.81±0.08 | 0.29±0.17 | 1 |
|  |  | GB | 0.54±0.11 | 0.58±0.12 | 0.67±0.09 | 0.4±0.24 | 1 |
| BI | PD | SVM-lin | 0.5±0.05 | 0.51±0.1 | 0.89±0.04 | 0.11±0.09 | 1 |
|  |  | SVM-rbf | 0.5±0.05 | 0.48±0.1 | 0.92±0.07 | 0.07±0.07 | 1 |
|  |  | GB | 0.48±0.09 | 0.47±0.1 | 0.64±0.09 | 0.32±0.18 | 1 |
|  | 1 year | SVM-lin | 0.49±0.1 | 0.51±0.25 | 0.95±0.05 | 0.04±0.2 | 1 |
|  |  | SVM-rbf | 0.52±0.13 | 0.47±0.26 | 0.96±0.05 | 0.08±0.27 | 1 |
|  |  | GB | 0.42±0.08 | 0.41±0.27 | 0.84±0.16 | 0.0±0.0 | 1 |
| TICS-M | 1 year | SVM-lin | 0.58±0.07 | 0.66±0.09 | 0.85±0.08 | 0.32±0.15 | 0,495 |
|  |  | SVM-rbf | 0.56±0.08 | 0.64±0.1 | 0.84±0.08 | 0.28±0.15 | 0,885 |
|  |  | GB | 0.6±0.08 | 0.66±0.1 | 0.72±0.09 | 0.48±0.16 | 0 |
|  | 3 years | SVM-lin | 0.65±0.13 | 0.8±0.1 | 0.87±0.07 | 0.42±0.25 | 0,27 |
|  |  | SVM-rbf | 0.63±0.13 | 0.79±0.11 | 0.89±0.07 | 0.37±0.25 | 0,465 |
|  |  | GB | 0.61±0.16 | 0.64±0.17 | 0.71±0.11 | 0.5±0.3 | 0,57 |
| MMSE | PD | SVM-lin | 0.57±0.07 | 0.65±0.1 | 0.9±0.06 | 0.23±0.15 | 1 |
|  |  | SVM-rbf | 0.59±0.08 | 0.67±0.09 | 0.93±0.06 | 0.25±0.15 | 0,195 |
|  |  | GB | 0.56±0.08 | 0.58±0.09 | 0.69±0.08 | 0.43±0.18 | 1 |
| CES-D | 1 year | SVM-lin | 0.53±0.07 | 0.58±0.1 | 0.85±0.08 | 0.22±0.12 | 1 |
|  |  | SVM-rbf | 0.53±0.06 | 0.59±0.09 | 0.89±0.08 | 0.17±0.12 | 1 |
|  |  | GB | 0.48±0.08 | 0.53±0.09 | 0.63±0.1 | 0.34±0.15 | 1 |
|  | 3 years | SVM-lin | 0.47±0.07 | 0.47±0.1 | 0.84±0.11 | 0.09±0.11 | 1 |
|  |  | SVM-rbf | 0.49±0.04 | 0.47±0.11 | 0.92±0.11 | 0.06±0.11 | 1 |
|  |  | GB | 0.53±0.1 | 0.51±0.13 | 0.67±0.12 | 0.39±0.2 | 1 |
| Survival | 1 year | SVM-lin | 0.51±0.05 | 0.58±0.1 | 0.91±0.05 | 0.11±0.11 | 1 |
|  |  | SVM-rbf | 0.51±0.05 | 0.58±0.11 | 0.95±0.05 | 0.08±0.1 | 1 |
|  |  | GB | 0.45±0.07 | 0.42±0.09 | 0.65±0.11 | 0.25±0.14 | 1 |
|  | 3 years | SVM-lin | 0.51±0.05 | 0.52±0.07 | 0.42±0.08 | 0.6±0.09 | 1 |
|  |  | SVM-rbf | 0.5±0.05 | 0.46±0.05 | 0.23±0.16 | 0.76±0.18 | 1 |
|  |  | GB | 0.51±0.04 | 0.52±0.05 | 0.26±0.13 | 0.76±0.13 | 1 |

Abbreviations: mRS, Modified Rankin Scale; BI, Barthel Index; MMSE, Mini-Mental State Examination; TICS-M, Modified Telephone Interview for Cognitive Status; CES-D, Epidemiologic Studies Depression Scale; SVM-lin, Support Vector Machine with linear kernel; SVM-rbf, Support Vector Machine with radial basis function kernel; GB, Gradient Boosting Classifier; PD, Patient discharge; BA, Balance accuracy; AUC, Area under the curve; SD, Standard deviation

Table S5. Results for the serological input subdomain and all models listed in BA±SD, AUC±SD, Sensitivity±SD, Specificity±SD and p-values according to the permutation test.

| **Labels** | **Time** | **Model** | **BA±SD** | **AUC±SD** | **Sensitivity±SD** | **Specificity±SD** | **P-value** |
| --- | --- | --- | --- | --- | --- | --- | --- |
| mRS | PD | SVM-lin | 0.58±0.06 | 0.61±0.07 | 0.7±0.08 | 0.45±0.12 | 0,27 |
|  |  | SVM-rbf | 0.57±0.06 | 0.63±0.07 | 0.77±0.09 | 0.37±0.14 | 0,015 |
|  |  | GB | 0.63±0.07 | 0.68±0.08 | 0.69±0.08 | 0.56±0.13 | 0 |
|  | 1 year | SVM-lin | 0.64±0.09 | 0.66±0.11 | 0.71±0.06 | 0.58±0.19 | 0,06 |
|  |  | SVM-rbf | 0.63±0.1 | 0.64±0.12 | 0.69±0.07 | 0.58±0.19 | 0 |
|  |  | GB | 0.6±0.09 | 0.69±0.09 | 0.74±0.07 | 0.45±0.18 | 0,195 |
| BI | PD | SVM-lin | 0.57±0.07 | 0.61±0.1 | 0.71±0.06 | 0.43±0.14 | 1 |
|  |  | SVM-rbf | 0.57±0.08 | 0.6±0.1 | 0.72±0.08 | 0.42±0.16 | 1 |
|  |  | GB | 0.57±0.08 | 0.62±0.09 | 0.73±0.06 | 0.41±0.17 | 0,48 |
|  | 1 year | SVM-lin | 0.54±0.19 | 0.66±0.19 | 0.87±0.12 | 0.2±0.4 | 1 |
|  |  | SVM-rbf | 0.61±0.2 | 0.63±0.25 | 0.84±0.15 | 0.38±0.49 | 1 |
|  |  | GB | 0.51±0.16 | 0.45±0.28 | 0.9±0.1 | 0.12±0.33 | 1 |
| TICS-M | 1 year | SVM-lin | 0.61±0.08 | 0.69±0.08 | 0.73±0.08 | 0.49±0.14 | 0,09 |
|  |  | SVM-rbf | 0.61±0.09 | 0.67±0.09 | 0.73±0.08 | 0.49±0.15 | 0,12 |
|  |  | GB | 0.6±0.08 | 0.65±0.09 | 0.74±0.09 | 0.46±0.16 | 0,135 |
|  | 3 years | SVM-lin | 0.62±0.12 | 0.61±0.15 | 0.72±0.08 | 0.51±0.24 | 0,915 |
|  |  | SVM-rbf | 0.62±0.13 | 0.65±0.15 | 0.73±0.12 | 0.52±0.25 | 0,165 |
|  |  | GB | 0.55±0.1 | 0.57±0.13 | 0.79±0.09 | 0.3±0.21 | 1 |
| MMSE | PD | SVM-lin | 0.59±0.09 | 0.67±0.07 | 0.7±0.06 | 0.49±0.18 | 0,705 |
|  |  | SVM-rbf | 0.58±0.09 | 0.65±0.07 | 0.66±0.06 | 0.51±0.19 | 0,36 |
|  |  | GB | 0.55±0.11 | 0.6±0.11 | 0.76±0.07 | 0.35±0.23 | 1 |
| CES-D | 1 year | SVM-lin | 0.6±0.07 | 0.6±0.09 | 0.7±0.08 | 0.49±0.15 | 0,21 |
|  |  | SVM-rbf | 0.58±0.08 | 0.59±0.09 | 0.71±0.1 | 0.45±0.16 | 0,195 |
|  |  | GB | 0.58±0.1 | 0.62±0.1 | 0.7±0.08 | 0.46±0.17 | 0,645 |
|  | 3 years | SVM-lin | 0.55±0.11 | 0.56±0.11 | 0.67±0.11 | 0.43±0.23 | 1 |
|  |  | SVM-rbf | 0.54±0.1 | 0.56±0.11 | 0.65±0.13 | 0.44±0.23 | 1 |
|  |  | GB | 0.51±0.11 | 0.54±0.11 | 0.72±0.1 | 0.29±0.2 | 1 |
| Survival | 1 year | SVM-lin | 0.48±0.07 | 0.46±0.1 | 0.66±0.07 | 0.29±0.14 | 1 |
|  |  | SVM-rbf | 0.49±0.08 | 0.48±0.11 | 0.67±0.09 | 0.3±0.16 | 1 |
|  |  | GB | 0.54±0.1 | 0.59±0.1 | 0.76±0.07 | 0.32±0.18 | 1 |
|  | 3 years | SVM-lin | 0.56±0.05 | 0.6±0.06 | 0.46±0.09 | 0.66±0.07 | 0,75 |
|  |  | SVM-rbf | 0.53±0.04 | 0.61±0.06 | 0.31±0.14 | 0.76±0.11 | 0,75 |
|  |  | GB | 0.57±0.06 | 0.61±0.05 | 0.54±0.15 | 0.61±0.12 | 0,09 |

Abbreviations: mRS, Modified Rankin Scale; BI, Barthel Index; MMSE, Mini-Mental State Examination; TICS-M, Modified Telephone Interview for Cognitive Status; CES-D, Epidemiologic Studies Depression Scale; SVM-lin, Support Vector Machine with linear kernel; SVM-rbf, Support Vector Machine with radial basis function kernel; GB, Gradient Boosting Classifier; PD, Patient discharge; BA, Balance accuracy; AUC, Area under the curve; SD, Standard deviation

Table S6. Results for the MRI input subdomain and all models listed in BA±SD, AUC±SD, Sensitivity±SD, Specificity±SD and p-values according to the permutation test.

| **Labels** | **Time** | **Model** | **BA±SD** | **AUC±SD** | **Sensitivity±SD** | **Specificity±SD** | **P-value** |
| --- | --- | --- | --- | --- | --- | --- | --- |
| mRS | PD | SVM-lin | 0.54±0.06 | 0.6±0.08 | 0.89±0.06 | 0.2±0.09 | 1 |
|  |  | SVM-rbf | 0.5±0.03 | 0.58±0.08 | 0.92±0.07 | 0.09±0.07 | 1 |
|  |  | GB | 0.52±0.06 | 0.56±0.07 | 0.7±0.07 | 0.33±0.09 | 1 |
|  | 1 year | SVM-lin | 0.52±0.05 | 0.55±0.12 | 0.95±0.04 | 0.08±0.09 | 1 |
|  |  | SVM-rbf | 0.5±0.03 | 0.52±0.1 | 0.98±0.04 | 0.02±0.05 | 1 |
|  |  | GB | 0.51±0.08 | 0.56±0.09 | 0.73±0.09 | 0.3±0.14 | 1 |
| BI | PD | SVM-lin | 0.54±0.05 | 0.57±0.12 | 0.95±0.03 | 0.12±0.09 | 1 |
|  |  | SVM-rbf | 0.53±0.04 | 0.61±0.11 | 0.97±0.03 | 0.1±0.09 | 1 |
|  |  | GB | 0.54±0.07 | 0.58±0.1 | 0.72±0.08 | 0.36±0.14 | 1 |
|  | 1 year | SVM-lin | 0.5±0.0 | 0.35±0.24 | 1.0±0.01 | 0.0±0.0 | 1 |
|  |  | SVM-rbf | 0.5±0.01 | 0.35±0.26 | 1.0±0.01 | 0.0±0.0 | 1 |
|  |  | GB | 0.49±0.14 | 0.44±0.22 | 0.88±0.12 | 0.1±0.3 | 1 |
| TICS-M | 1 year | SVM-lin | 0.53±0.06 | 0.55±0.1 | 0.9±0.08 | 0.15±0.1 | 1 |
|  |  | SVM-rbf | 0.52±0.06 | 0.55±0.12 | 0.94±0.07 | 0.11±0.11 | 1 |
|  |  | GB | 0.51±0.08 | 0.55±0.1 | 0.7±0.11 | 0.33±0.15 | 1 |
|  | 3 years | SVM-lin | 0.49±0.03 | 0.46±0.11 | 0.97±0.04 | 0.01±0.05 | 1 |
|  |  | SVM-rbf | 0.49±0.02 | 0.4±0.14 | 0.98±0.04 | 0.0±0.04 | 1 |
|  |  | GB | 0.53±0.11 | 0.57±0.14 | 0.81±0.1 | 0.25±0.23 | 1 |
| MMSE | PD | SVM-lin | 0.55±0.06 | 0.68±0.09 | 0.96±0.03 | 0.14±0.13 | 1 |
|  |  | SVM-rbf | 0.53±0.06 | 0.68±0.09 | 0.96±0.04 | 0.09±0.14 | 1 |
|  |  | GB | 0.56±0.1 | 0.62±0.11 | 0.75±0.09 | 0.36±0.25 | 1 |
| CES-D | 1 year | SVM-lin | 0.55±0.07 | 0.53±0.12 | 0.92±0.05 | 0.18±0.14 | 1 |
|  |  | SVM-rbf | 0.56±0.07 | 0.53±0.12 | 0.94±0.05 | 0.19±0.14 | 0,78 |
|  |  | GB | 0.56±0.1 | 0.56±0.12 | 0.74±0.08 | 0.38±0.2 | 0,825 |
|  | 3 years | SVM-lin | 0.48±0.04 | 0.49±0.11 | 0.93±0.06 | 0.03±0.08 | 1 |
|  |  | SVM-rbf | 0.48±0.03 | 0.5±0.14 | 0.95±0.06 | 0.01±0.03 | 1 |
|  |  | GB | 0.47±0.1 | 0.47±0.14 | 0.7±0.12 | 0.24±0.19 | 1 |
| Survival | 1 year | SVM-lin | 0.49±0.01 | 0.5±0.11 | 0.98±0.02 | 0.0±0.02 | 1 |
|  |  | SVM-rbf | 0.5±0.01 | 0.35±0.09 | 0.99±0.01 | 0.0±0.0 | 1 |
|  |  | GB | 0.48±0.08 | 0.4±0.11 | 0.81±0.07 | 0.13±0.14 | 1 |
|  | 3 years | SVM-lin | 0.5±0.05 | 0.51±0.06 | 0.33±0.09 | 0.67±0.11 | 1 |
|  |  | SVM-rbf | 0.49±0.03 | 0.48±0.05 | 0.14±0.13 | 0.85±0.15 | 1 |
|  |  | GB | 0.5±0.05 | 0.51±0.06 | 0.23±0.2 | 0.77±0.2 | 1 |

Abbreviations: mRS, Modified Rankin Scale; BI, Barthel Index; MMSE, Mini-Mental State Examination; TICS-M, Modified Telephone Interview for Cognitive Status; CES-D, Epidemiologic Studies Depression Scale; SVM-lin, Support Vector Machine with linear kernel; SVM-rbf, Support Vector Machine with radial basis function kernel; GB, Gradient Boosting Classifier; PD, Patient discharge; BA, Balance accuracy; AUC, Area under the curve; SD, Standard deviation

Table S7. Comparison of all models using all input variables with Integrated Discrimination Improvement (IDI) and Likelihood Ratio (LR) with p-values.

| **Outcome** | **Time point** | **Model 1** | **Model 2** | **IDI** | **LR** | **P-value** |
| --- | --- | --- | --- | --- | --- | --- |
| mRS | PD | SVC-lin | SVC-rbf | -0,13 | -0,4 | 1 |
|  |  | SVC-lin | GB | -0,13 | -0,36 | 1 |
|  |  | SVC-rbf | GB | 0 | 0,04 | 1 |
|  | 1 year | SVC-lin | SVC-rbf | -0,06 | -0,64 | 1 |
|  |  | SVC-lin | GB | -0,13 | -0,58 | 1 |
|  |  | SVC-rbf | GB | -0,07 | 0,06 | 1 |
| BI | PD | SVC-lin | SVC-rbf | 0 | -0,4 | 1 |
|  |  | SVC-lin | GB | -0,04 | -0,24 | 1 |
|  |  | SVC-rbf | GB | -0,04 | 0,16 | 1 |
|  | 1 year | SVC-lin | SVC-rbf | 0,24 | -0,18 | 1 |
|  |  | SVC-lin | GB | 0,11 | -0,2 | 1 |
|  |  | SVC-rbf | GB | -0,17 | -0,02 | 1 |
| MMSE | PD | SVC-lin | SVC-rbf | 0 | -0,66 | 1 |
|  |  | SVC-lin | GB | 0,15 | -0,46 | 1 |
|  |  | SVC-rbf | GB | 0,15 | 0,2 | 1 |
| TICS-M | 1 year | SVC-lin | SVC-rbf | 0,03 | -1,36 | 1 |
|  |  | SVC-lin | GB | 0 | -1,32 | 1 |
|  |  | SVC-rbf | GB | -0,03 | 0,04 | 1 |
|  | 3 years | SVC-lin | SVC-rbf | 0,08 | -0,56 | 1 |
|  |  | SVC-lin | GB | 0,12 | -0,58 | 1 |
|  |  | SVC-rbf | GB | 0,04 | -0,02 | 1 |
| CES-D | 1 year | SVC-lin | SVC-rbf | -0,06 | -0,7 | 1 |
|  |  | SVC-lin | GB | -0,12 | -0,64 | 1 |
|  |  | SVC-rbf | GB | -0,06 | 0,06 | 1 |
|  | 3 years | SVC-lin | SVC-rbf | 0,06 | -2,36 | 1 |
|  |  | SVC-lin | GB | 0,2 | -2,22 | 1 |
|  |  | SVC-rbf | GB | 0,15 | 0,14 | 1 |
| Survival | 1 year | SVC-lin | SVC-rbf | -0,04 | -0,54 | 1 |
|  |  | SVC-lin | GB | 0,1 | -0,24 | 1 |
|  |  | SVC-rbf | GB | 0,13 | 0,3 | 1 |
|  | 3 years | SVC-lin | SVC-rbf | 0,1 | -0,38 | 1 |
|  |  | SVC-lin | GB | -0,15 | -0,42 | 1 |
|  |  | SVC-rbf | GB | -0,28 | -0,04 | 1 |

Abbreviations: mRS, Modified Rankin Scale; BI, Barthel Index; MMSE, Mini-Mental State Examination; TICS-M, Modified Telephone Interview for Cognitive Status; CES-D, Epidemiologic Studies Depression Scale; SVM-lin, Support Vector Machine with linear kernel; SVM-rbf, Support Vector Machine with radial basis function kernel; GB, Gradient Boosting Classifier; PD, Patient discharge; IDI, Integrated discrimination improvement; LR, Likelihood Ratio

Table S8. Comparison of all models using the demographic input subdomain with Integrated Discrimination Improvement (IDI) and Likelihood Ratio (LR) with p-values.

| **Outcome** | **Time point** | **Model 1** | **Model 2** | **IDI** | **LR** | **P-value** |
| --- | --- | --- | --- | --- | --- | --- |
| mRS | PD | SVC-lin | SVC-rbf | 0 | -0,02 | 1 |
|  |  | SVC-lin | GB | 0,07 | 0 | 1 |
|  |  | SVC-rbf | GB | 0,07 | 0,02 | 1 |
|  | 1 year | SVC-lin | SVC-rbf | 0 | -0,02 | 1 |
|  |  | SVC-lin | GB | 0,21 | 0,06 | 1 |
|  |  | SVC-rbf | GB | 0,21 | 0,08 | 1 |
| BI | PD | SVC-lin | SVC-rbf | 0,02 | -0,02 | 1 |
|  |  | SVC-lin | GB | 0,07 | -0,02 | 1 |
|  |  | SVC-rbf | GB | 0,05 | 0 | 1 |
|  | 1 year | SVC-lin | SVC-rbf | 0,18 | 0 | 1 |
|  |  | SVC-lin | GB | 0,36 | 0 | 1 |
|  |  | SVC-rbf | GB | 0,23 | 0 | 1 |
| MMSE | PD | SVC-lin | SVC-rbf | -0,03 | 0 | 1 |
|  |  | SVC-lin | GB | 0,09 | -0,04 | 1 |
|  |  | SVC-rbf | GB | 0,12 | -0,04 | 1 |
| TICS-M | 1 year | SVC-lin | SVC-rbf | 0,04 | 0 | 1 |
|  |  | SVC-lin | GB | 0,13 | 0,04 | 1 |
|  |  | SVC-rbf | GB | 0,1 | 0,04 | 1 |
|  | 3 years | SVC-lin | SVC-rbf | 0,04 | -0,02 | 1 |
|  |  | SVC-lin | GB | 0,12 | -0,06 | 1 |
|  |  | SVC-rbf | GB | 0,08 | -0,04 | 1 |
| CES-D | 1 year | SVC-lin | SVC-rbf | -0,07 | 0 | 1 |
|  |  | SVC-lin | GB | -0,07 | -0,02 | 1 |
|  |  | SVC-rbf | GB | 0 | -0,02 | 1 |
|  | 3 years | SVC-lin | SVC-rbf | -0,05 | -0,06 | 1 |
|  |  | SVC-lin | GB | -0,05 | -0,1 | 1 |
|  |  | SVC-rbf | GB | 0 | -0,04 | 1 |
| Survival | 1 year | SVC-lin | SVC-rbf | 0,04 | -0,02 | 1 |
|  |  | SVC-lin | GB | -0,09 | -0,04 | 1 |
|  |  | SVC-rbf | GB | -0,13 | -0,02 | 1 |
|  | 3 years | SVC-lin | SVC-rbf | 0,13 | 0 | 1 |
|  |  | SVC-lin | GB | -0,02 | -0,02 | 1 |
|  |  | SVC-rbf | GB | -0,18 | -0,02 | 1 |

Abbreviations: mRS, Modified Rankin Scale; BI, Barthel Index; MMSE, Mini-Mental State Examination; TICS-M, Modified Telephone Interview for Cognitive Status; CES-D, Epidemiologic Studies Depression Scale; SVM-lin, Support Vector Machine with linear kernel; SVM-rbf, Support Vector Machine with radial basis function kernel; GB, Gradient Boosting Classifier; PD, Patient discharge; IDI, Integrated discrimination improvement; LR, Likelihood Ratio

Table S9. Comparison of all models using the clinical input subdomain with Integrated Discrimination Improvement (IDI) and Likelihood Ratio (LR) with p-values.

| **Outcome** | **Time point** | **Model 1** | **Model 2** | **IDI** | **LR** | **P-value** |
| --- | --- | --- | --- | --- | --- | --- |
| mRS | PD | SVC-lin | SVC-rbf | -0,02 | -0,02 | 1 |
|  |  | SVC-lin | GB | 0,14 | 0,08 | 1 |
|  |  | SVC-rbf | GB | 0,16 | 0,1 | 1 |
|  | 1 year | SVC-lin | SVC-rbf | 0,03 | 0 | 1 |
|  |  | SVC-lin | GB | 0,24 | 0,18 | 1 |
|  |  | SVC-rbf | GB | 0,21 | 0,18 | 1 |
| BI | PD | SVC-lin | SVC-rbf | 0,06 | -0,04 | 1 |
|  |  | SVC-lin | GB | 0,08 | 0,22 | 1 |
|  |  | SVC-rbf | GB | 0,02 | 0,26 | 1 |
|  | 1 year | SVC-lin | SVC-rbf | 0,08 | -0,02 | 1 |
|  |  | SVC-lin | GB | 0,17 | 0,16 | 1 |
|  |  | SVC-rbf | GB | 0,1 | 0,18 | 1 |
| MMSE | PD | SVC-lin | SVC-rbf | -0,06 | -0,04 | 1 |
|  |  | SVC-lin | GB | 0,17 | 0,36 | 1 |
|  |  | SVC-rbf | GB | 0,21 | 0,4 | 1 |
| TICS-M | 1 year | SVC-lin | SVC-rbf | 0,06 | 0 | 1 |
|  |  | SVC-lin | GB | 0 | 0,04 | 1 |
|  |  | SVC-rbf | GB | -0,06 | 0,04 | 1 |
|  | 3 years | SVC-lin | SVC-rbf | 0,05 | -0,04 | 1 |
|  |  | SVC-lin | GB | 0,44 | 0,34 | 1 |
|  |  | SVC-rbf | GB | 0,42 | 0,38 | 1 |
| CES-D | 1 year | SVC-lin | SVC-rbf | -0,02 | -0,04 | 1 |
|  |  | SVC-lin | GB | 0,12 | 0,1 | 1 |
|  |  | SVC-rbf | GB | 0,15 | 0,14 | 1 |
|  | 3 years | SVC-lin | SVC-rbf | 0 | -0,16 | 1 |
|  |  | SVC-lin | GB | -0,08 | 0 | 1 |
|  |  | SVC-rbf | GB | -0,08 | 0,16 | 1 |
| Survival | 1 year | SVC-lin | SVC-rbf | 0 | -0,04 | 1 |
|  |  | SVC-lin | GB | 0,28 | 0,38 | 1 |
|  |  | SVC-rbf | GB | 0,28 | 0,42 | 1 |
|  | 3 years | SVC-lin | SVC-rbf | 0,11 | -0,06 | 1 |
|  |  | SVC-lin | GB | 0 | -0,06 | 1 |
|  |  | SVC-rbf | GB | -0,12 | 0 | 1 |

Abbreviations: mRS, Modified Rankin Scale; BI, Barthel Index; MMSE, Mini-Mental State Examination; TICS-M, Modified Telephone Interview for Cognitive Status; CES-D, Epidemiologic Studies Depression Scale; SVM-lin, Support Vector Machine with linear kernel; SVM-rbf, Support Vector Machine with radial basis function kernel; GB, Gradient Boosting Classifier; PD, Patient discharge; IDI, Integrated discrimination improvement; LR, Likelihood Ratio

Table S10. Comparison of all models using the serological input subdomain with Integrated Discrimination Improvement (IDI) and Likelihood Ratio (LR) with p-values.

| **Outcome** | **Time point** | **Model 1** | **Model 2** | **IDI** | **LR** | **P-value** |
| --- | --- | --- | --- | --- | --- | --- |
| mRS | PD | SVC-lin | SVC-rbf | -0,05 | -0,1 | 1 |
|  |  | SVC-lin | GB | -0,22 | -0,16 | 1 |
|  |  | SVC-rbf | GB | -0,16 | -0,06 | 1 |
|  | 1 year | SVC-lin | SVC-rbf | 0,06 | -0,04 | 1 |
|  |  | SVC-lin | GB | -0,1 | -0,2 | 1 |
|  |  | SVC-rbf | GB | -0,16 | -0,16 | 1 |
| BI | PD | SVC-lin | SVC-rbf | 0,03 | -0,06 | 1 |
|  |  | SVC-lin | GB | -0,03 | -0,14 | 1 |
|  |  | SVC-rbf | GB | -0,05 | -0,08 | 1 |
|  | 1 year | SVC-lin | SVC-rbf | 0,08 | 0,02 | 1 |
|  |  | SVC-lin | GB | 0,38 | -0,14 | 1 |
|  |  | SVC-rbf | GB | 0,33 | -0,16 | 1 |
| MMSE | PD | SVC-lin | SVC-rbf | 0,06 | -0,02 | 1 |
|  |  | SVC-lin | GB | 0,18 | -0,2 | 1 |
|  |  | SVC-rbf | GB | 0,13 | -0,18 | 1 |
| TICS-M | 1 year | SVC-lin | SVC-rbf | 0,06 | -0,08 | 1 |
|  |  | SVC-lin | GB | 0,11 | -0,12 | 1 |
|  |  | SVC-rbf | GB | 0,06 | -0,04 | 1 |
|  | 3 years | SVC-lin | SVC-rbf | -0,11 | -0,4 | 1 |
|  |  | SVC-lin | GB | 0,09 | -0,5 | 1 |
|  |  | SVC-rbf | GB | 0,19 | -0,1 | 1 |
| CES-D | 1 year | SVC-lin | SVC-rbf | 0,02 | -0,06 | 1 |
|  |  | SVC-lin | GB | -0,05 | -0,1 | 1 |
|  |  | SVC-rbf | GB | -0,08 | -0,04 | 1 |
|  | 3 years | SVC-lin | SVC-rbf | 0 | -0,16 | 1 |
|  |  | SVC-lin | GB | 0,04 | -0,16 | 1 |
|  |  | SVC-rbf | GB | 0,04 | 0 | 1 |
| Survival | 1 year | SVC-lin | SVC-rbf | -0,04 | -0,1 | 1 |
|  |  | SVC-lin | GB | -0,32 | -0,26 | 1 |
|  |  | SVC-rbf | GB | -0,27 | -0,16 | 1 |
|  | 3 years | SVC-lin | SVC-rbf | -0,03 | -0,02 | 1 |
|  |  | SVC-lin | GB | -0,03 | -0,04 | 1 |
|  |  | SVC-rbf | GB | 0 | -0,02 | 1 |

Abbreviations: mRS, Modified Rankin Scale; BI, Barthel Index; MMSE, Mini-Mental State Examination; TICS-M, Modified Telephone Interview for Cognitive Status; CES-D, Epidemiologic Studies Depression Scale; SVM-lin, Support Vector Machine with linear kernel; SVM-rbf, Support Vector Machine with radial basis function kernel; GB, Gradient Boosting Classifier; PD, Patient discharge; IDI, Integrated discrimination improvement; LR, Likelihood Ratio

Table S11. Comparison of all models using the MRI input subdomain with Integrated Discrimination Improvement (IDI) and Likelihood Ratio (LR) with p-values.

| **Outcome** | **Time point** | **Model 1** | **Model 2** | **IDI** | **LR** | **P-value** |
| --- | --- | --- | --- | --- | --- | --- |
| mRS | PD | SVC-lin | SVC-rbf | 0,05 | 0 | 1 |
|  |  | SVC-lin | GB | 0,09 | 0,08 | 1 |
|  |  | SVC-rbf | GB | 0,05 | 0,08 | 1 |
|  | 1 year | SVC-lin | SVC-rbf | 0,06 | -0,12 | 1 |
|  |  | SVC-lin | GB | -0,02 | 0,14 | 1 |
|  |  | SVC-rbf | GB | -0,09 | 0,26 | 1 |
| BI | PD | SVC-lin | SVC-rbf | -0,1 | -0,1 | 1 |
|  |  | SVC-lin | GB | -0,02 | 0,3 | 1 |
|  |  | SVC-rbf | GB | 0,07 | 0,4 | 1 |
|  | 1 year | SVC-lin | SVC-rbf | 0 | -0,08 | 1 |
|  |  | SVC-lin | GB | -0,16 | 0,3 | 1 |
|  |  | SVC-rbf | GB | -0,16 | 0,38 | 1 |
| MMSE | PD | SVC-lin | SVC-rbf | 0 | -0,08 | 1 |
|  |  | SVC-lin | GB | 0,16 | 0,32 | 1 |
|  |  | SVC-rbf | GB | 0,16 | 0,4 | 1 |
| TICS-M | 1 year | SVC-lin | SVC-rbf | 0 | -0,1 | 1 |
|  |  | SVC-lin | GB | 0 | 0,02 | 1 |
|  |  | SVC-rbf | GB | 0 | 0,12 | 1 |
|  | 3 years | SVC-lin | SVC-rbf | 0,1 | -0,14 | 1 |
|  |  | SVC-lin | GB | -0,26 | 0,1 | 1 |
|  |  | SVC-rbf | GB | -0,4 | 0,24 | 1 |
| CES-D | 1 year | SVC-lin | SVC-rbf | 0 | -0,16 | 1 |
|  |  | SVC-lin | GB | -0,07 | 0,02 | 1 |
|  |  | SVC-rbf | GB | -0,07 | 0,18 | 1 |
|  | 3 years | SVC-lin | SVC-rbf | -0,02 | -0,14 | 1 |
|  |  | SVC-lin | GB | 0,04 | 0,1 | 1 |
|  |  | SVC-rbf | GB | 0,06 | 0,24 | 1 |
| Survival | 1 year | SVC-lin | SVC-rbf | 0,23 | -0,08 | 1 |
|  |  | SVC-lin | GB | 0,17 | 0,34 | 1 |
|  |  | SVC-rbf | GB | -0,08 | 0,42 | 1 |
|  | 3 years | SVC-lin | SVC-rbf | 0,06 | -0,06 | 1 |
|  |  | SVC-lin | GB | 0 | -0,04 | 1 |
|  |  | SVC-rbf | GB | -0,06 | 0,02 | 1 |

Abbreviations: mRS, Modified Rankin Scale; BI, Barthel Index; MMSE, Mini-Mental State Examination; TICS-M, Modified Telephone Interview for Cognitive Status; CES-D, Epidemiologic Studies Depression Scale; SVM-lin, Support Vector Machine with linear kernel; SVM-rbf, Support Vector Machine with radial basis function kernel; GB, Gradient Boosting Classifier; PD, Patient discharge; IDI, Integrated discrimination improvement; LR, Likelihood Ratio
